# The correlation of sacral table angle to spinopelvic sagittal alignment in healthy adults

**DOI:** 10.1186/s13018-023-03782-w

**Published:** 2023-04-24

**Authors:** Nan Ru, Keith D. K. Luk, Jianmin Sun, Guodong Wang

**Affiliations:** 1 Department of Spine Surgery, Shandong Provincial Hospital affiliated to Shandong First Medical University, No. 9677, Jingshi Road, Jinan, Shandong Province China; 2 Department of Bone and Joint Surgery and Sports Medicine Center, The First Affiliated Hospital, Jinan University, Guangzhou, China; 3 Orthopedics and Sports Medicine Center, The Hong Kong Sanatorium and Hospitals, Happy Valley, Hong Kong SAR China

**Keywords:** Spinopelvic sagittal alignment, Predictive models, Sacral table angle, Sacral inclination, Pelvic incidence

## Abstract

**Background:**

The sacrum plays an important role in sagittal balance of the spine, whereas the exact association between sacral parameters, specifically the sacral table angle (STA) and spinopelvic parameters has been only scarcely assessed. It aims to investigate the correlations between the sacral parameters and spinopelvic sagittal alignment parameters in healthy adults.

**Methods:**

A cohort of 142 Northern Chinese healthy adults between 18 and 45 years old were recruited between April 2019 and March 2021. Full-spine standing X-ray films were performed for every volunteer. The sacral parameters were measured: sacral table angle (STA), sacral inclination (SI) and sacral slope (SS). The spinopelvic sagittal alignment parameters included: pelvic incidence (PI), pelvic tilt (PT), lumbar lordosis (LL), thoracic kyphosis and the apex of lumbar lordosis (LLA). The correlations analysis, as well as the linear regression analysis, were performed between STA, SI and the spinopelvic parameters.

**Results:**

An equation ‘STA = SI + 90 − SS’ was revealed to represent the interrelationships between STA, SI and SS. STA was statistically correlated with PI (*r*_*s*_ = − 0.693), PT (*r*_*s*_ = − 0.342), SS (*r*_*s*_ = − 0.530), LL (*r*_*s*_ = 0.454), and LLA (*r*_*s*_ = 0.438). SI correlated with STA (*r*_*s*_ = 0.329), PT (*r*_*s*_ =  − 0.562), SS (*r*_*s*_ =  − 0.612) and LL (*r*_*s*_ = 0.476). Simple linear regression analysis also verified the correlation between STA and PI (*y* = − 1.047*x* + 149.4), SS (*y* = − 0.631*x* + 96.9), LL (*y* = 0.660*x* − 117.7), LLA (*y* = 0.032*x* + 0.535), and SI (*y* = 0.359*x* + 8.23).

**Conclusion:**

The equation ‘STA = SI + 90 − SS’ indicates the exact geometric relationship between STA, SI and SS. The sacral parameters, both STA and SI, correlate to the spinopelvic sagittal alignment parameters in healthy adults. The linear regression analysis results also give predictive models for spinopelvic sagittal alignment parameters based on the invariant parameter STA, which are helpful for surgeons in designing an ideal therapeutic plan.

## Background

It is the spinal sagittal balance rather than the coronal balance to be significantly correlated with health-related quality of life (HRQOL) [1]. The reconstruction of the sagittal balance is supposed as a major issue for successful long-term outcome in spinal surgery [2]. Numerous spinal sagittal parameters have been proposed to assess the spinal sagittal balance [3, 4].

The concept of ‘pelvic vertebra’ is a cornerstone in the study of sagittal balance [5]. The pelvic morphology further determines the spinal sagittal alignment [6, 7]. The pelvic ring consists of the hip bones and the sacrum. Thus, the sacral morphology has an important influence on both pelvic morphology and spinal morphology. Several studies have elaborated the potential role of sacral morphology on the spinal sequence [8–10].

**Fig. 1:**
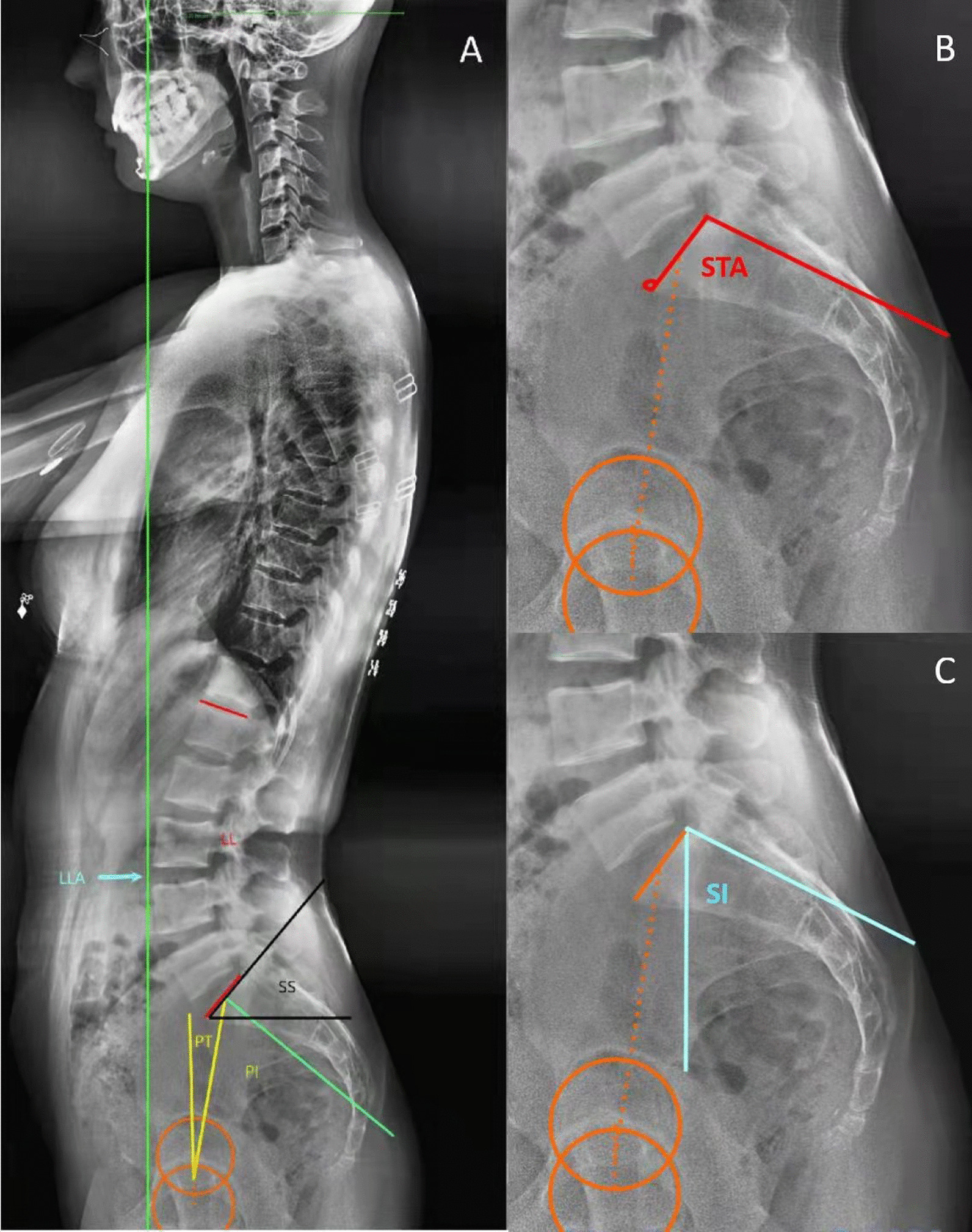
The descriptions of spinopelvic and sacral parameters. *PI* Pelvic incidence; *PT* Pelvic tilt; *SS* Sacral slope; *LL* Lumbar lordosis; *LLA* The apex of lumbar lordosis; *SI* Sacral inclination; *STA* Sacral table angle

There are two groups of sacral parameters: sacral slope (SS) and sacral inclination (SI) are ‘positional’ parameters which can change with posture, whereas pelvic incidence (PI) and sacral table angle (STA) are fixed bony ‘anatomical’ parameters. (Fig. 1) [11, 12]. Previous studies have demonstrated that STA played an important role in the complex spinopelvic interaction and the development of spinal degeneration [8, 13]. Sacral slope is a positional parameter of sacrum which has been studied widely. Sacral inclination (SI) is the other representant positional parameter of sacrum, which was used to describe lumbar spondylolisthesis [14]. However, the exact relationships between STA, SI and SS, as well as the other spinopelvic sagittal alignments in healthy adults have not been studied systematically.

The focus of the study is on STA which was not well studied in the past and to find out its correlation with the other sacral positional parameters and spinopelvic sagittal alignments parameters in healthy adults, which would provide information helpful for future surgical planning for patients.

## Materials and methods

### Patient population

A cohort of 158 Northern Chinese healthy adults between 18 and 45 years old was recruited between April 2019 and March 2021. Out of the 158 healthy volunteers, 142 healthy volunteers were enrolled in the study. Written informed consent was obtained from all subjects who participated in this study, and ethical approval was provided by the local relevant committee.

The exclusion criteria were as follows: (1) lumbopelvic transitional vertebrae, (2) spinal deformity or spondylolisthesis, (3) lumbar or thoracic disease, (4) hip joint or pelvic disease, (5) history of prior spinal surgery, (6) neurological or neuromuscular disease and (7) pregnancy.

### Radiographic measurements of sagittal parameters

Full-spine (posterior–anterior and lateral) radiographs were collected from all volunteers in a standardized standing position [15] and unclear images were excluded. All radiographic parameters were measured by Surgimap software, version 2.3.1.5 (Nemaris, Inc., New York, USA).

The following sagittal parameters were measured according to the convention in the literature [3, 6, 12, 16]: spinopelvic sagittal parameters, including PI, PT, SS, LL, thoracic kyphosis (TK) and the apex of lumbar lordosis (LLA); and sacral parameters, including STA and SI. STA was defined the angle between the sacral endplate and posterior border of the S1 body. And SI was defined the angle between the vertical plane and the posterior border of the S1 body. The detailed measurement methods of these above sagittal parameters are exhibited graphically in Fig. 1 and Table 1.

**Table 1:** The measurements of sagittal parameters

Parameters	Measurements
PI	The angle between the line perpendicular to the sacral plate at its midpoint and the line connecting this point to the femoral head axis
PT	The angle between the vertical line and the line joining the middle of the sacral plate and the hip axis
SS	The angle between the sacral endplate and the horizontal line
LL	The angle between the superior endplate of S1 and L1
TK	The angle between the upper endplate of T4 and lower endplate of T12
LLA	The LLA was defined as the most anterior lumbar vertebra or disc in the sagittal plane. Vertebrae from L1 to L5 were assigned numbers ranging from 1 to 5 to simplify data collection as well as to facilitate correlation analysis
SI	The angle between the vertical plane and the posterior border of the S1 body
STA	The angle between the sacral endplate and posterior border of the S1 body
SVA	The offset between the centre of C7 and the plumb line drawn from posterosuperior corner of S1

*PI* Pelvic incidence; *PT* Pelvic tilt; *SS* Sacral slope; *LL* Lumbar lordosis; *TK* Thoracic kyphosis; *LLA* The apex of lumbar lordosis; *SI* Sacral inclination; *STA* Sacral table angle

All radiographic parameters were measured twice by two independent experienced clinicians, and the average value was calculated as the final result for the following analysis. The intra- and inter-observer variability was evaluated by the intra-class correlation coefficient (ICC) in all subjects. The results showed that the intra-observer ICCs for PI, PT, SS, LL, TK, LLA, STA and SI were 0.956, 0.991, 0.979, 0.982, 0.969, 0.977, 0.989 and 0.984, respectively, while the inter-observer ICCs were 0.977, 0.968, 0.979, 0.958, 0.972, 0.964, 0.970 and 0.956, respectively. Based on the Shrout and Fleiss criteria for reliability testing, both the intra- and inter-observer reproducibility were excellent [4].

### Statistical analysis

All statistical analyses were performed using SPSS software 21.0 (SPSS Inc., Chicago, Illinois). The normality of the data was first tested by the Shapiro–Wilk test and all the parameters were expressed as the mean ± SD (standard deviation). The correlations between spinal sagittal parameters, SI and STA, were analysed using the Pearson or Spearman correlation coefficient, and simple linear regressions were conducted when significant correlations were identified. The statistical significance threshold was *P* < 0.05.

## Results

A total of 142 adults (females and males) with a mean age of 31.0 ± 9.7 years (range 18–45 years) participated in the present study. The descriptive statistics and a spectrum of the normal variations in the spinal sagittal parameters are detailed in Table 2.

**Table 2:** Description of spinal sagittal radiographic parameters

Parameters	Mean	SD	Min	Max
PI (°)	43.6	9.0	20.0	68.3
PT (°)	10.5	7.2	− 9.2	31.7
SS (°)	33.1	7.1	17.5	51.4
LL (°)	− 51.1	9.1	− 77	− 26
TK (°)	25.5	8.8	6.6	50.3
LLA	3.8	0.4	2.5	5
SI (°)	44.5	6.5	26.7	62.0
STA (°)	101.0	6.0	85.1	118.4
SVA (cm)	− 2.9	2.9	− 4.6	5.0

*PI* Pelvic incidence; *PT* Pelvic tilt; *SS* Sacral slope; *LL* Lumbar lordosis; *TK* Thoracic kyphosis; *LLA* The apex of lumbar lordosis; *SI* Sacral inclination; *STA* Sacral table angle

**Fig. 2:**
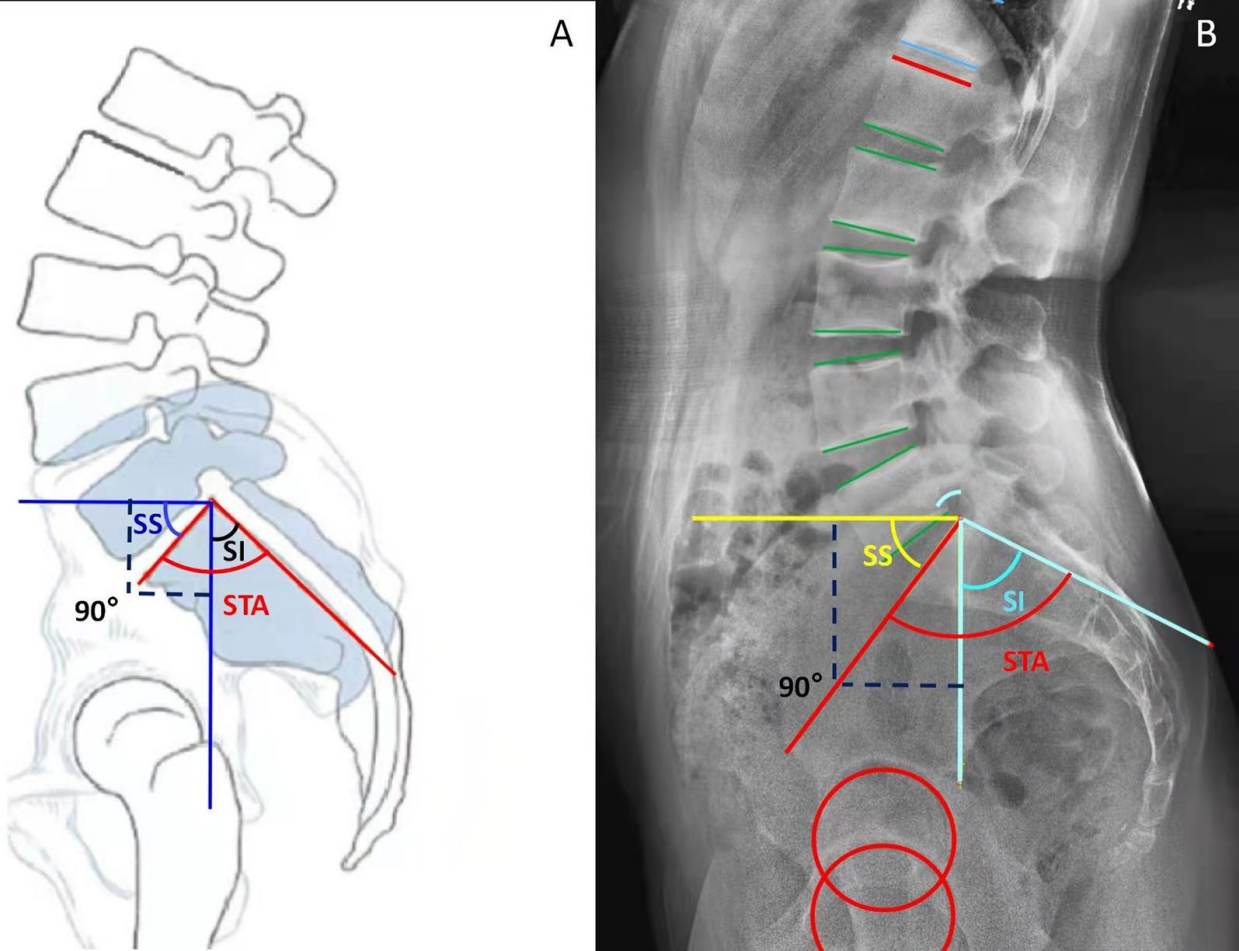
The schematic diagram shows the geometric formula equation between STA, SI, and SS: ‘STA = SI + 90 − SS’. *STA* Sacral table angle; *SI* Sacral inclination; *SS* Sacral slope

**Fig. 3:**
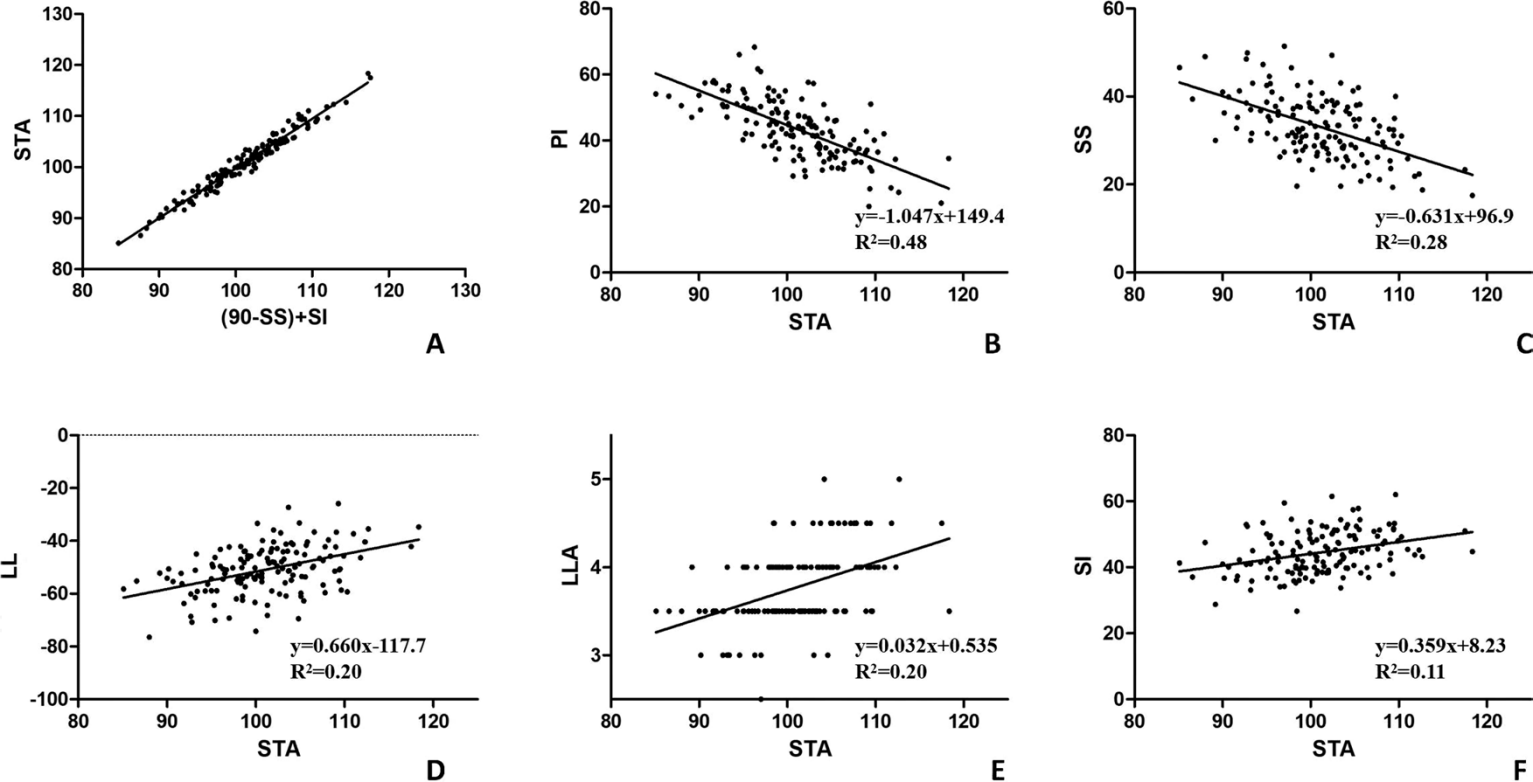
**A** linear regression between (SI + 90 − SS) and STA; **B**–**F**, linear correlations between sacral table angle and spinopelvic sagittal alignment parameters. *SI* Sacral inclination; *SS* Sacral slope; *STA* Sacral table angle; *PI* Pelvic incidence; *PT* Pelvic tilt; *LL* Lumbar lordosis; *LLA* The apex of lumbar lordosis; *SI* Sacral inclination

In this study, geometric construction by complementary angles revealed one ingenious equation, that is STA = SI + 90 − SS (Fig. 2). The equation was also verified by correlation analysis and simple linear regression analysis (Fig. 3A). In the formula, the pelvic positional parameter, the sacral positional parameter and the sacral anatomical parameters were combined for analysis.

As for the correlation between STA, SI and other sagittal parameters. The results showed that STA was statistically correlated with PI, SI, PT, SS, LL and LLA; but not with age, TK or SVA. The detailed associations are shown in Table 3. Simple linear regression analysis also verified the correlation between STA and PI (*y* = − 1.047*x* + 149.4, *R*^2^ = 0.48, *P* < 0.001), SS (*y* = − 0.631*x* + 96.9, *R*^2^ = 0.28, *P* < 0.001), LL (*y* = 0.660*x *− 117.7, *R*^2^ = 0.20, *P* < 0.001), LLA (*y* = 0.032*x* + 0.535, *R*^2^ = 0.20, *P* < 0.001), and SI (*y* = 0.359*x* + 8.23, *R*^2^ = 0.11, *P* < 0.001). The detailed corresponding linear regressions are summarized in Fig. 3.

**Table 3:** Correlations between spinal sagittal parameters and sacral table angle

Spinal sagittal parameters	Correlation coefficient	*P* value
Age (years)	0.017	0.840
PI (°)	− 0.693	< 0.001*
PT (°)	− 0.342	< 0.001*
SS (°)	− 0.530	< 0.001*
LL (°)	0.454	< 0.001*
LLA	0.438	< 0.001*
TK (°)	0.135	0.108
SVA (cm)	− 0.062	0.461

*PI* Pelvic incidence; *PT* Pelvic tilt; *SS* Sacral slope; *LL* Lumbar lordosis; *TK* Thoracic kyphosis

*With significance

**Fig. 4:**
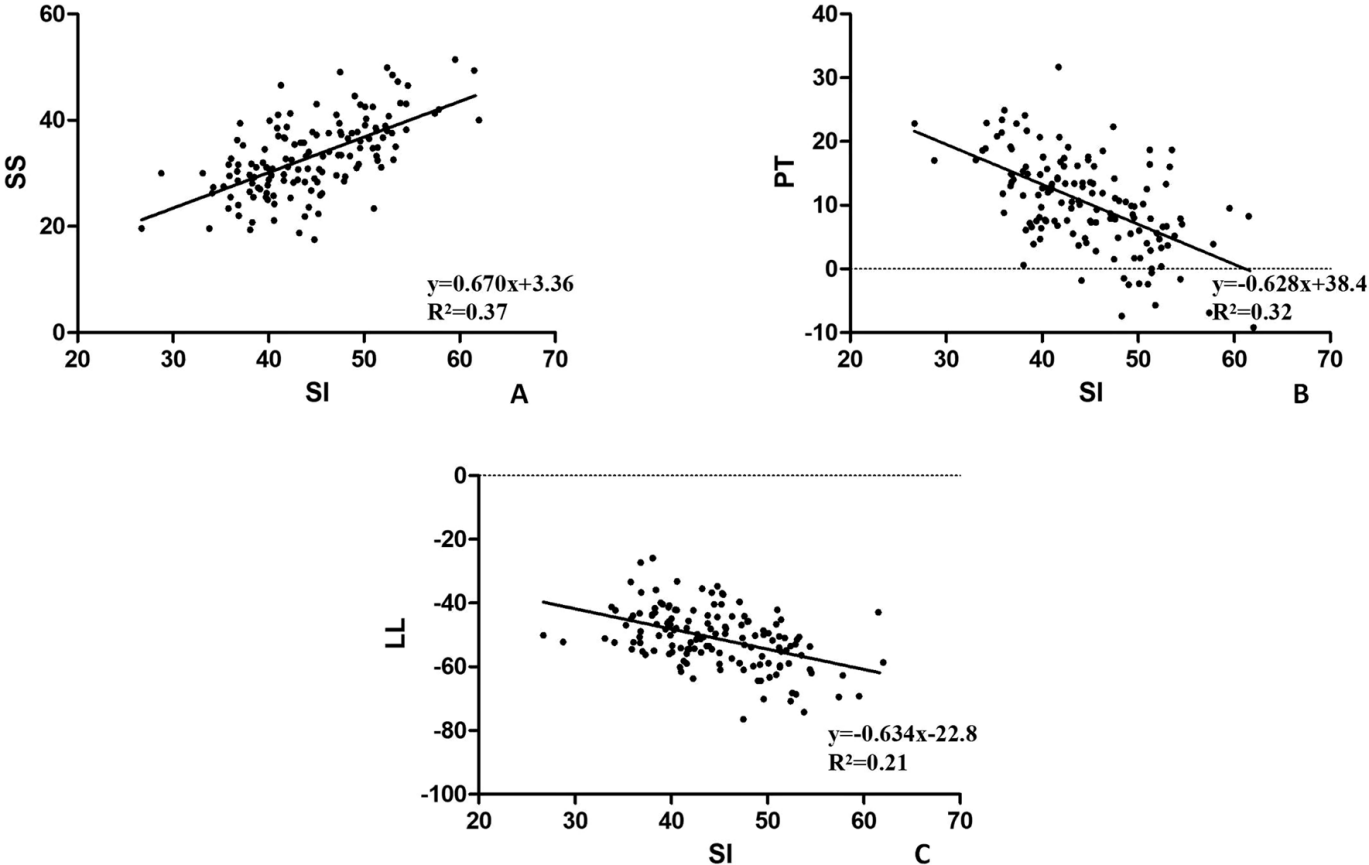
Linear correlations between the sacral inclination and spinopelvic sagittal alignment parameters. *SI* Sacral inclination; *SS* Sacral slope; *PT* Pelvic tilt; *LL* Lumbar lordosis

Also, SI was statistically correlated with PT (*r* = − 0.562, *P* < 0.001), SS (*r* = − 0.612, *P* < 0.001), LL (*r* = 0.476, *P* < 0.001) and TK (*r* = 0.190, *P* = 0.024); but not with age, PI, LLA or SVA. The detailed associations are shown in Table 4. Simple linear regression analysis also verified the correlation between SI and PT (*y* = − 0.628*x* + 38.4, *R*^2^ = 0.32, *P* < 0.001), SS (*y* = 0.670*x* + 3.36, *R*^2^ = 0.37, *P* < 0.001) and LL (*y* = − 0.634*x*− 22.8, *R*^2^ = 0.21, *P* < 0.001). The details are shown in Fig. 4.

**Table 4:** Correlations between spinal sagittal parameters and sacral inclination

Spinal sagittal parameters	Correlation coefficient	*P* value
Age (years)	− 0.164	0.051
STA (°)	0.329	< 0.001*
PI (°)	0.030	0.719
PT (°)	− 0.562	< 0.001*
SS (°)	− 0.612	< 0.001*
LL (°)	0.476	< 0.001*
LLA	− 0.014	0.868
TK (°)	0.190	0.024*
SVA (cm)	0.007	0.937

*STA* Sacral table angle; *PI* Pelvic incidence; *PT* Pelvic tilt; *SS* sacral slope; *LL* Lumbar lordosis; *LLA* The apex of lumbar lordosis; *TK* Thoracic kyphosis

*With significance

## Discussion

The pelvis was the pedal of spine, which was regarded as the ‘pelvic vertebra’ by Jean Dubousset [5]. The shape and the orientation of pelvis were determined inherently by gene expression. It further influenced the sequential alignments of the whole spine [17]. Legaye et al. introduced PI to describe the morphology of the pelvis, and PT and SS to describe the orientation of the pelvis [17]. There was a classical geometric equation ‘PI = PT + SS’ proposed simultaneously.

On the basis of these three pelvic parameters, numerous spinopelvic parameters such as global tilt (GT), T1-pelvic angle (TPA), LLA, LLLA and inflexion point (IP) had been proposed to better describe the sagittal balance of spine[4, 18, 19]. The good correlation between PI and the parameters aforementioned further verified the cornerstone role of pelvic morphology in the study of sagittal balance. Even though the important role of sacral morphology in maintaining spinal sagittal balance cannot be ignored, many previous studies had also confirmed this viewpoint. However, the exact association between sacral parameters and spinopelvic parameters in healthy adults has not been studied systematically.

In the present study, we selected STA, a characteristic sacral parameter for correlation analysis. We found the STA strongly and negatively correlated with PI (*r* = − 0.714). That is, a large PI is accompanied by a small STA, and a small PI was associated with a large STA [11]. Previous investigations have demonstrated that patients with large STA were more susceptible to lumbar disc herniation, whereas patients with small STA were prone to lumbar degenerative spondylolisthesis [20, 21]. Ergun had also reported that the degree and risk of intervertebral disc degeneration and herniation increases in parallel to the increase of STA in the case of the same PI [22]. However, these STA-related studies concentrated on its difference in different spinal diseases but failed to explore the exact association between STA and other sagittal parameters. In this study, we found that the STA showed high significant correlations with spinal sagittal parameters, especially the lumbar parameters in healthy adults, which filled this gap in the literature. We found that a large STA was accompanied by a flat and short lumbar curvature, a lower apex of lumbar lordosis and a horizontal sacral plateau, whereas a small STA was accompanied by a curved and long lumbar curvature, a higher apex of lumbar lordosis and an inclined sacral plateau. As is well known, Roussouly et al. introduced four types of lumbar lordosis in a normal adult population, and each type possessed a distinct morphological characteristic and degenerative pattern. Previous studies have verified that Type 2 lordosis (small PI, flat and short LL, low LLA and small SS) was prone to lumbar disc herniation, whereas Type 4 lordosis (large PI, curved and long LL, high LLA and large SS) was prone to lumbar spondylolisthesis [23]. These results are mutually verified with the result of Strube. Strube considered that a large STA results in a small SS by making the sacrum plateau more horizontal and further promotes disc degeneration.

The corresponding linear regressions we established have important clinical relevance and implications. Based on the invariant characteristic of STA, by virtue of the predictive formulas we established, the spinal surgeon could obtain the reference values of lumbopelvic sagittal parameters. The predictive formulae based on STA have the following advantages: (i) Currently, almost all the predictive formulas are based on PI, and the measurement of PI must be accurate. However, in some cases with aspherical femoral heads, with subluxation of the hip, and with osteoarthritis of the hip, failure to clearly identify the femoral head may cause large measurement errors of PI. In these cases, STA could be a good substitute for PI to obtain the reference values of lumbopelvic sagittal parameters. (ii) STA can be measured accurately and easily, which bring great convenience for clinical decision-making.

SI was defined as the angle between the vertical plane and the posterior border of the S1 body [14]. It was a sacral positional parameter and used to describe the rotation of the sacrum in lumbar spondylolisthesis. The sacroiliac joint was almost immobilized and thus the sacral positional parameter can represent pelvic positional parameters.

To date, only one previous study had investigated the correlation between SI, LL and PI [24]. In this study, we comprehensively analysed the correlations between SI and spinopelvic alignments. The correlation analysis showed that SI was strongly correlated with lumbopelvic parameters especially positional parameters in healthy adults (SS, *r* = − 0.612; PT, *r* = − 0.562). These findings could help us to quickly obtain the lumbar morphology though SI also has unique strengths: (i) the posterior border of the S1 body can be easily identify and is not readily deformed compared to the sacral plateau when L5S1 segment degeneration. (ii) SI is a very intuitive metric; it can be measured from the body surface by non-invasive and demonstrate good agreement with X-ray measurements [25, 26].

In addition, we attempted to explore the normal range of SI values in Chinese adults.

Thus far, no relevant studies have been reported. In this study, we found that SI was highly variable in Chinese healthy individuals (range from 26.7 to 62.0). The distribution of SI was similar to that of Turks (range from 32.0 to 70.0) and Indians (range from 35.0 to 62.0) [24, 25], whereas the distribution of SI in Germans shows larger fluctuation (range from 27.0 to 95.0) [26], which demonstrated that sacral parameters varies among different ethnic groups.

Finally, we established an exact geometric relationship between STA, SI and SS. The geometric formula equation is STA = SI + 90 − SS, in which the sacral parameters were bundled together. The fixed sacral anatomical parameter (STA) was artificially divided into two positional parameters (SI and SS). While STA represented the inherent sacral shape, SI and SS indicated a horizontal or vertical sacrum. The latter ones were common in degenerative spinal diseases, such as spondylolisthesis and adult degenerative spinal deformity. The equation ‘STA = SI + 90 − SS’ is similar to the equation ‘PI = SS + PT’. SS was included in both the equations. It combined the sacral parameters and pelvic parameters, reflected the tight integration of the sacrum and pelvis. Through this formula, it is easy to anticipate the position and shape of the pelvis by sacral parameters.

Although there are many significant results in this study, some limitations must be mentioned. First, we only evaluated the correlation between STA and spinopelvic parameters from the perspective of imaging, further anatomical evidence is needed to clarify what causes the difference in STA. To our knowledge, no relevant anatomical studies have been reported. Second, in this cross-sectional study, it seems difficult to investigate the effect of sacral morphology on spinal degeneration patterns. Long-term follow-up of these healthy volunteers is planned to explore more details about the impact of sacral morphology on spinal degeneration.

## Conclusion

In summary, the equation ‘STA = SI + 90 − SS’ indicates the exact geometric relationship between STA, SI and SS. It bundles together the sacral anatomical parameter (STA) and the positional parameters (SI and SS). The sacral anatomical parameters, both STA and SI, correlate to the spinopelvic sagittal alignment parameters in healthy adults. The linear regression analysis results also give predictive models for spinopelvic sagittal alignment parameters based on the invariant STA.

## Data Availability

The datasets analysed in the current study are available from the corresponding author on reasonable request.

## Abbreviations

SI: Sacral inclination
SS: Sacral slope
STA: Sacral table angle
PI: Pelvic incidence
PT: Pelvic tilt
LL: Lumbar lordosis
LLA: The apex of lumbar lordosis
SI: Sacral inclination
